# Young Adult German Breast Cancer Patients Participating in a Three-Week Inpatient Mother–Child Rehab Program Have High Needs for Supportive Care

**DOI:** 10.3390/cancers15061770

**Published:** 2023-03-15

**Authors:** Friederike Hammersen, Dorothea Fischer, Telja Pursche, Angelika M. Strobel, Alexander Katalinic, Louisa Labohm, Annika Waldmann

**Affiliations:** 1 Institute for Social Medicine and Epidemiology, University of Luebeck, Ratzeburger Allee 160, 23562 Luebeck, Germany; alexander.katalinic@uksh.de (A.K.); louisa.labohm@uksh.de (L.L.); 2 Department of Obstetrics and Gynecology, Ernst von Bergmann Clinic, Charlottenstraße 72, 14467 Potsdam, Germany; dorothea.fischer@klinikumevb.de; 3 Department of Gynecology and Obstetrics, Hospital Dueren gem. GmbH, Roonstraße 30, 52351 Dueren, Germany; telja.pursche@krankenhaus-dueren.de; 4 Department of Obstetrics and Gynecology, University Hospital of Schleswig-Holstein, Campus Luebeck, Ratzeburger Allee 160, 23562 Luebeck, Germany; 5 Institute for Cancer Epidemiology e.V., University of Luebeck, Ratzeburger Allee 160, 23562 Luebeck, Germany

**Keywords:** breast neoplasms, young adults, minor children, need for supportive care, quality of life, oncology, rehabilitation, parental cancer, EORTC QLQ-C30, follow-up

## Abstract

**Simple Summary:**

Breast cancer may lead to a reduced quality of life. Certain aspects such as sleep disorders and pain may be a problem not only in the short but also in the long term. Only recently have thresholds for quality of life scores indicating a need for supportive care been published. We asked 853 women with young children and with a breast cancer diagnosis before the age of 40 years about their quality of life, using a questionnaire called EORTC QLQ-C30. More than half of the women with breast cancer reported a reduced quality of life requiring supportive care. This was true 1 year and several years after diagnosis. Physicians, nurses, and other persons working in healthcare should (1) be aware of this and (2) talk to breast cancer survivors about supportive care options.

**Abstract:**

A known cut-off problem hampers the interpretation of quality of life (QOL) scores. The purpose of this study was to apply a novel approach for the EORTC QLQ-C30 instrument to identify the proportion of breast cancer (BC) patients in need of supportive care. Changes in QOL during the COVID-19 pandemic were evaluated, as well as changes over time (after treatment termination and up to 4 years later). Data were obtained from a cohort study on young adult BC patients with minor children participating in a mother–child rehab program. Cross-sectional QOL data were collected from 2015 to 2021 (baseline). Follow-up data were available for up to 4 years after diagnosis for a subgroup. The baseline cohort included 853 women (mean age 35 years). More than 50% had a need for supportive care. In the subgroup with follow-up, this proportion remained at a high level up to several years after diagnosis. During the COVID-19 pandemic, changes regarding the proportion with this need were not as high as expected—with the exception of changes on the QLQ-C30 scale ‘role functioning’ (+15%). Even several years after diagnosis, every second BC patient with minor children had a need for supportive care, which is much higher than previously found. Healthcare staff should be aware of this potential need and should address this issue.

## 1. Introduction

The mean age of female breast cancer (BC) diagnosis in Germany is 64 years [1] and for a long time, studies on BC mainly had a focus on this older age group or age-mixed cohorts [2]. However, breast cancer in young women is rare, as one out of six women with BC falls ill under the age of 50 and one out of twenty under the age of 40 years [3]. Following the European Society of Breast Cancer Specialists’ (EUSOMA) definition, young breast cancer patients are often defined as <40 years at diagnosis [4]. Young BC patients differ in many aspects from older BC patients; they are often affected by more aggressive tumours in advanced stages with worse prognoses [5,6,7,8]. They typically hold a different position in society, which is characterized by occupation and family life. With a long period of (working) life still ahead, the quality of life (QOL) of these young BC survivors is of major importance. Compared to older BC patients, younger patients are characterized by an impaired QOL in terms of depressiveness, sleep problems, fatigue, and fear of recurrence as well as poor body image, disruptions in relationships, occupational difficulties, and financial toxicity [6,9]. Studies have found associations between a worse QOL and higher needs, e.g., for psychosocial support [10,11,12,13,14].

In patient care, clinicians need one fast tool to determine which patients need further attention with regard to their QOL and in which aspects of QOL (psychosocial, daily activities, etc.). Thus, clinicians require a cut-off to determine which QOL scores lie outside the normal range of a patient’s QOL and which changes over time are meaningful. A commonly used QOL questionnaire among cancer patients is the European Organisation for Research and Treatment of Cancer Questionnaire-Core 30 (EORTC QLQ-C30) [15]. Different approaches have been suggested to find suitable cut-offs for this questionnaire, [16,17,18,19,20,21,22]. With the aim of focusing on the individual patient, Snyder et al. [20,21] identified EORCT QLQ-C30 scores that require the attention of a physician in terms of unmet patient needs. Their first study included patients with different cancer entities (mean age: 61 years). Their replication study included BC patients (mean age: 56 years). They were able to identify scores for six of the 15 EORTC QLQ-C30 domains [20,21]. Giesinger et al. [18] pursued another approach and a different research aim; they described thresholds of clinical importance for each scale of the EORTC QLQ-C30 based on patients with different cancer entities (mean age: 60 years).

Given the large differences between younger and older patients with regard to clinical characteristics, treatments, QOL, and their societal roles [5,6,7,8], it remained questionable whether the thresholds derived from older and age-mixed cohorts, respectively, would apply to younger patients as well. Only recently, Lidington et al. [22] reported cut-off scores for nine EORTC QLQ-C30 scales that indicate the need for supportive care in young adults with cancer (age range: 25 to 39 years). Using a very similar method to Snyder et al. [20,21], they found quite similar cut-off scores in general, but a remarkably lower score for emotional functioning. In addition, they were able to identify cut-offs for three further scales [22].

The onset of the COVID-19 pandemic represented an exceptional situation and affected essential areas of daily life, the economy, and supportive and healthcare [23,24,25] as well as mental health [26]. In the German healthcare system, resources were prioritized for potential, very sick COVID-19 patients [27], which led to changes in the oncological care in Germany—mainly in follow-up care, psycho-oncologic care, and surgery [28,29]. Among cancer patients, more than every second person showed symptoms of anxiety and every third reported symptoms of depression during the pandemic [27]. Those with high levels of anxiety reported barriers to contact healthcare providers and treatment delays [30,31].

For about a decade, our research group has been running a prospective cohort study on young adult women with BC participating in a 3-week inpatient mother–child rehabilitation program [5]. Applying the age range and the thresholds described by Lidington et al. to our study, we aim to describe the proportion with need for supportive care in a population of young adult BC patients with minor children, who participate in a 3-week inpatient rehabilitation program. Furthermore, we aim to display time trends of these proportions up to 4 years after diagnosis and aim to assess whether these trends changed with the onset of the COVID-19 pandemic.

## 2. Materials and Methods

### 2.1. Cohort Study in the Context of the Rehab Program “Get Well Together”

Data on QOL were obtained from a prospective cohort study embedded in the context of a rehabilitation program. Women with BC can participate in this 3-week inpatient mother–child rehabilitation program named “get well together” (in German: “gemeinsam gesund werden”) in Groemitz, Northern Germany. The rehab program focusses on women with BC who are mothers to at least one child under the age of 12 years who accompanies them. The children benefit from special group activities led by a psychologist, focusing, e.g., on coping strategies and empowerment. Inclusion criteria for the rehab program are a diagnosis of BC (ICD-10 C50) or ductal carcinoma in situ (DCIS, ICD-10 D05.1), completed primary treatment, and no signs of distant metastases.

Upon arrival in the rehab clinic, the women are consecutively invited to participate in our study. They independently fill out a study questionnaire during their rehab stay. From an earlier recruitment period (2010 to 2011; cohort 2), we know that the response rate among the rehab patients was high (85%).

About 500 women form a cohort and their study questionnaires cover cohort-specific topics (e.g., genetic counselling in cohort 4, physical activity in cohort 5, psychosocial health of children in cohort 6). Clinical characteristics, oncological care, and QOL belong to the core modules of the study questionnaire, which are repeated in every cohort. In the year 2018, we conducted a follow-up survey with participants from cohorts 2 to 4 who consented to receive self-administered follow-up questionnaires. The follow-up survey focused on the course of disease and quality of life.

Our cohort study is registered with the German Register for Clinical Studies (www.dkrs.de; accessed on 10 November 2022; registry number DKRS00025060). For more information on the cohort study, see [5,32,33,34].

### 2.2. Quality of Life

QOL was assessed using the German version of the European Organisation for Research and Treatment of Cancer Quality of Life Questionnaire-Core 30 (EORTC QLQ-C30; version 3.0). This is a widely used questionnaire that has been validated in different patient groups and many languages [15,35]. The EORTC QLQ-C30 comprises 30 items divided among five functional scales (physical, role, cognitive, emotional, and social functioning), nine symptom scales/items (fatigue, nausea/vomiting, pain, dyspnoea, insomnia, appetite loss, constipation, diarrhoea, financial difficulties), and a global health/QOL scale. The raw scores are transformed into a score range from 0 to 100. Higher scores on the functioning scales and the global health status/QOL indicate a higher quality of life. The symptom scales are reversed, meaning that higher scores indicate more physical restraints [36].

### 2.3. Need for Supportive Care

Lidington et al. [22] identified cut-offs for the EORTC QLC-C30 scales to indicate which young adult cancer patients are probably in need of supportive care (age range: 25–39 years). Therefore, they allocated relevant items from the Supportive Care Needs Survey (SCNS-LF59) to each EORTC QLQ-C30 scale. In this context, the need for supportive care is broadly defined as the perceived needs of people diagnosed with cancer. The SCNS-LF59 covers different need domains: psychological needs, health system and information needs, physical and daily living needs, patient care and support needs, and sexuality needs as well as four additional items. Lidington et al. proposed cut-offs with adequate sensitivity (i.e., >0.7) for nine EORTC QLQ-C30 scales, namely for global health status/QOL, for four functional (physical, role, emotional, and social functioning) and for four symptom scales/items (fatigue, nausea and vomiting, pain, and insomnia) [22].

### 2.4. Clinical Characteristics, Treatment, and Socio-Demographics

Patient files provided information on clinical characteristics and tumour treatment. The classification of tumour biology was based on the 2011 St Gallen surrogate definition [37]. During the earliest rehab year included in the present analysis, data on systemic anticancer treatment (SACT; mainly chemotherapy) were only captured as “no”, “yes”, “yes, study medication”, and “unknown”, while later on, the main SACT agents were recorded and treatment was additionally classified as conform vs. non-conform with current guideline recommendations. During the course of study, SACT treatments categories were steadily amended.

Socio-demographics were derived from the study questionnaire. Education was grouped into three categories based on the German system: ‘low’—until grade 9 or 10 (compulsory); ‘intermediate’—until grade 10; ‘high’—until grade 12 or 13. Employment was categorized as ‘workers’, ‘employees/civil servants’, and ‘self-employed/freelancers’. Income was divided into three categories (EUR < 1500; EUR 1500–3000; EUR > 3000) and refers to the monthly net income of all household members after taxes and social contributions.

### 2.5. Statistical Analysis

For the purpose of this analysis, we included baseline data from women recruited between 2015 (cohort 4) and 2021 (cohort 6). Furthermore, we used data from the follow-up survey for cohort 4 in order to describe the QOL and proportions with need for supportive care after their rehab stay. Following the EUSOMA definition for young BC patients [4], we restricted our analysis to women < 40 years of age at diagnosis. Similar to Lidington et al. [22], we set the minimum age at diagnosis to 25 years.

We used means and standard deviations for describing the central tendency and spread of quantitative/numerical data (age at diagnosis, months between diagnosis and rehab stay, EORTC QLQ-C30 scales). Categorical data were described with absolute and relative frequencies (socio-demographics, tumour characteristics, treatments, need for supportive care). The sample description is split into three groups according to the time of rehab stay/study participation: two pre-pandemic periods from 2015 to 2017 and 2018 to March 2020, respectively, and one pandemic period from July 2020 to 2021. The gap between March and July 2020 was caused by the pandemic-related closure of the rehab clinic.

In order to describe temporal trends, the proportions of young adult BC patients with need for supportive care (according to the cut-offs from [22]) are presented by year of rehab stay and with 95% confidence intervals. The upper and lower limits of the confidence intervals were calculated according to Altman et al. [38]. The internal consistency of EORTC QLQ-C30 scales was described using Cronbach’s alpha for scales with at least three items and with the Spearman–Brown coefficient for scales with two items contributing to the scale [39].

The nature of the present analysis was predominantly exploratory. Nevertheless, we performed statistical tests on the results regarding the QOL scores and the proportion with need for supportive care. We used the chi-squared test for independent, nominal/ordinal data, and the McNemar test for paired, dichotomous data. Effect sizes were quantified using omega squared (QOL scores, independent observations), Cramer’s V (nominal, independent observations), and Cohen’s g (dichotomous, paired data). Effect sizes were interpreted as follows: Cohen’s g values in the range of 0.05 to <0.15 indicated a small, values of 0.15 to <0.25 a medium, and values ≥ 0.25 indicated a large effect (using the absolute values of Cohen’s g). For Cramer’s V, values in the range of 0.07 to <0.2 indicated a minimal, of 0.2 to 0.35 a medium, and values ≥ 0.35 a large effect (three groups). For omega squared, values < 0.02 indicated a very small, values in the range of 0.02 to <0.13 a small, values in the range of 0.13 to <0.26 a median, and values ≥ 0.26 a large effect.

Statistical analysis and data visualization were carried out using R version 4.1.0 (packages janitor, lubridate, tidyverse, ggplot2, finalfit, psych, MOTE, rcompanion). Analysis validation was carried out using SPSS version 23.

The analysis code can be found at https://osf.io/yvadt/ (accessed on 13 March 2023).

## 3. Results

### 3.1. Sample Selecetion

**Figure 1:**
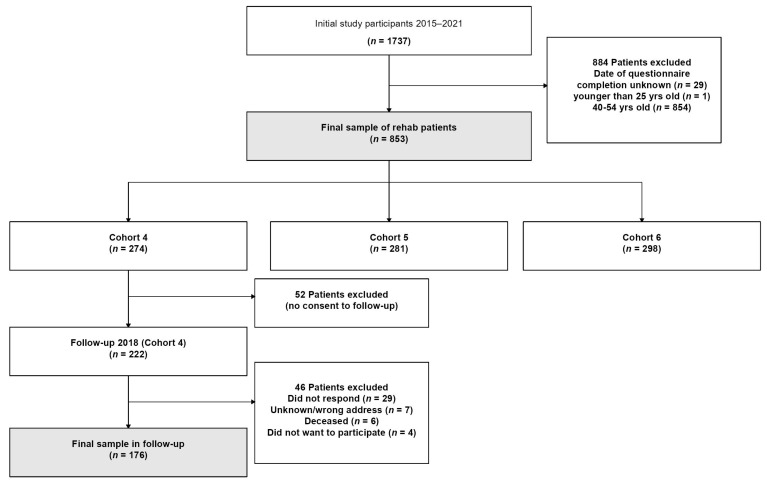
Flowchart of study sample for baseline survey and follow-up.

Overall, 1737 BC patients participated in our cohort study between 2015 and 2021, of which 29 women did not specify the exact date of completion of the questionnaire. In order to allow a presentation of QOL data by year of rehab, we excluded those women. A further 855 women were excluded because they were outside the age range of 25–39 years (Figure 1). The final sample size reduced to 853 young adult BC patients, of which about 16.6% had their rehab stay during the pandemic era (07/2020–2021).

### 3.2. Sample Description

The mean age at diagnosis was stable over time at about 35 years (Table 1). In general, women took part in the rehab program about 1 year after diagnosis. Based on self-reports, most young adult BC patients had a permanent partner and worked either as an employee or as a civil servant. The patients predominantly had an intermediate to high level of education and most often belonged to the highest income group. The proportion of patients in the highest income group increased over time.

### 3.3. Clinical Characteristics and Cancer Treatment

Data on clinical characteristics and treatment were extracted from the patient files in the rehab clinic. During the earliest time period, 2015 to 2017, proportionally, more tumours were diagnosed at larger tumour sizes (T2, T3) and more tumours had already spread into regional lymph nodes (N1–N3). Throughout the study period, high-grade tumours were predominant and luminal B/B1 and triple-negative were the most frequent tumour biology categories. About one third of the patients had a positive family anamnesis for BC (Table 2).

All patients had surgery, with breast-conserving treatment as the predominant surgery mode. Three out of 4 BC patients were treated with radiotherapy, about 2 out of 3 patients received hormone therapy, and 9 out of 10 patients received systemic anticancer treatment (chemotherapy). Less than 1 out of 3 patients with chemotherapy additionally received HER-2 targeted therapy. About 2 out of 3 patients received hormone therapy, mainly with selective oestrogen-receptor modulators (Table 3).

### 3.4. Need for Supportive Care

Self-reports on global health status/QOL and different aspects of QOL were quite stable when comparing the mean scores of subgroups according to rehab start. With the exception of the scales ‘physical function’ and ‘financial difficulties’, all tests for statistical significance yielded *p*-values > 0.5. All effect sizes were very small with omega squared = 0 (Supplementary Table S1). Measures of internal consistency for the EORTC QLQ-C30 scales were above 0.7 with one exception (scale ‘nausea and vomiting’, 0.67; Supplementary Table S2).

**Figure 2:**
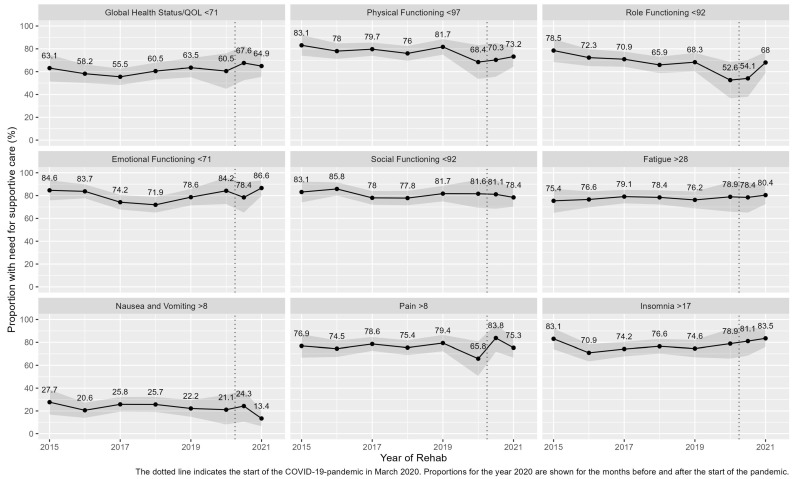
Proportions (and 95% confidence intervals) of young adult non-metastatic breast cancer patients with minor children participating in a 3-week inpatient mother–child rehab program with EORTC QLQ-C30 scores indicating a need for supportive care according to thresholds proposed by Lidington et al. [22] over time.

The proportions of patients with need for supportive care were also quite stable, as indicated by the proportions and their 95% confidence intervals (Figure 2). Deviating from these observations were the results for the scale ‘role functioning’ (see below).

With the exception of ‘nausea and vomiting’, more than half of the population and up to 86% were classified as having a “need for supportive care” irrespective of the time of rehab stay. The highest proportions of patients with need for supportive care could be found on the scales ‘emotional functioning’, ‘social functioning’, and ‘physical functioning’. Compared to the pre-pandemic years, the proportions with need for supportive care were higher during the pandemic era for ‘global health status/QOL’, ‘emotional functioning’ (in the year 2021), ‘fatigue’ (in the year 2021), and for ‘insomnia’, while the proportion with need for supportive care concerning ‘nausea and vomiting’ reached its lowest value in the year 2021. For ‘pain’, the proportion with need for supportive care reached a peak at the start of the pandemic era. For ‘role functioning’, the proportion with need for supportive care decreased by 25% until the start of COVID-19 pandemic, and then increased again by 15% until 2021.

### 3.5. Quality of Life and Need for Supportive Care during the Rehab Stay and Three Years Later

In total, 274 BC patients aged 25 to 39 years and with a rehab stay during the period from 2015 to 2017 were recruited as part of cohort 4. Among them, there were 222 who gave consent to further contact and these were invited for a follow-up survey in the year 2018. For a total of 176 patients, both the baseline and the follow-up questionnaires were available (Figure 1).

The proportions of BC patients with need for supportive care at baseline and at follow-up are shown in Table 4. Compared to baseline, fewer women were in need of supportive care at follow-up. The largest differences were seen for ‘role functioning’ (−17%; medium effect size with an absolute value of 0.21 for Cohen’s g), ‘physical functioning’ (−11%; Cohen’s g = 0.21), and ‘social functioning’ (−10%; Cohen’s g = 0.19). For the symptom scales/items, the differences were smaller and almost all effect sizes were small. The proportions with need for supportive care due to pain, fatigue, and sleep disturbance remained at a high level at follow-up (68–69%).

We further differentiated the 176 BC patients with baseline and follow-up information according to the time period between diagnosis and follow-up survey (≤24 months, 25 to 36 months, >36 months). A trend towards smaller proportions of women with need for supportive care was observed for the two groups, with longer time periods for nearly all QOL aspects compared to the period of ≤24 months between diagnosis and follow-up. In general, the differences were more pronounced between the groups ≤24 months and 25 to 36 months than the differences between the groups 25 to 36 months and >36 months between diagnosis and follow-up. Statistical tests yielded *p*-values > 0.05 and Cramer’s V indicated minimal effect sizes (all <0.2; Supplementary Table S3).

## 4. Discussion

In our prospective cohort study with 853 young adult non-metastatic BC patients with minor children who participated in a three-week inpatient mother–child rehabilitation program, we observed that more than every second patient had a need for supportive care. This was true not only 12 months after diagnosis, but also several years after diagnosis. We further observed for most QOL domains that during the COVID-19 pandemic, the proportions with need for supportive care among this population did not increase as much as could have been expected—with the exception of the scale role functioning, where the proportion with need for supportive care decreased over time until the start of COVID-19 pandemic, and increased with the onset of the pandemic.

### 4.1. Need for Supportive Care

In order to facilitate the usage and interpretation of QOL measures in clinical practice, several authors described cut-offs indicating value ranges that require the attention of healthcare staff. Only recently, Lidington et al. [22] published thresholds for the EORTC QLQ-C30 indicating a need for supportive care among young adult British cancer patients. When we applied these thresholds to our cohort of young adult German BC patients with minors, we observed that a high proportion of our cohort (>50%) had a need for supportive care. The only exception is the ‘nausea and vomiting’ scale, where less than 30% of our cohort scored values that indicate a need for supportive care. The results for our cohort by far exceed the proportions found by Lidington et al. [22], where proportions mostly ranged between 20% and 30%. ‘Emotional functioning’ showed the highest proportions in both populations, and was about 42% in Lidington et al.’s study and about twice as high in our study population. Although the sample of Lidington et al. is quite comparable to our sample in terms of sex, age, and cancer type, as it predominantly included females, predominantly with BC, and with an average age of 33 years at diagnosis, some discrepancies were observed. Surgery, chemo- and radiotherapy were more often performed in our cohort, which, amongst other factors, may have contributed to the higher needs for supportive care. Peters et al. [40] suggested that a combination of chemo- and radiotherapy could exacerbate cancer patients’ fatigue and hence also their physical functioning, which is in line with our results when compared to Lidington et al. [22]. In general, women seem to report a lower QOL than men [40]. Additionally, the importance of taking gender into account when interpreting QOL score has been described before [41]. As the thresholds for supportive care needs were derived from a gender-mixed cohort, this could have also contributed to our results with elevated proportions with need for supportive care. Additionally, all of our BC patients had at least one child under the age of 12 years, and previous studies showed that these patients have higher needs for psychosocial support than BC patients with older children and older BC patients, respectively [10]. In the general population, it was also found that parents in young adulthood were also more prone to mental health problems [26], which, in turn, could result in having a need for supportive care. Finally, we recruited our study population in the setting of an inpatient rehabilitation program. Therefore, it may be the case that our data are prone to “confounding by indication”. In Germany, each cancer patient with statutory health insurance is entitled to participate in an oncological rehab program [42]. However, only about 15% of patients with an incident cancer diagnosis participate in an oncological rehab [43]. The reasons for non-participation are manifold [44]. Among BC patients with minor children, a major obstacle is the question of who takes care of the children, as they usually do not accompany their mothers to rehab. Thus, it can be expected that this population takes part in rehab even less. The rehab program “get well together” aims to overcome this obstacle as women should be accompanied by at least one child under the age of 12 years. Therefore, it is conceivable that—given their specific position in society, which is characterized by occupation and family life—on the one hand, our young BC patients with minor children are more likely to have a need for supportive care compared to older BC patients and that on the other hand, especially those with a high need for supportive care seek to participate in an oncological rehab program. Taken together, these factors could be considered as “selection bias” and could explain why we observed a higher proportion with need for supportive care than has been described in the literature [22].

### 4.2. Need for Supportive Care and Changes Associated with the COVID-19 Pandemic (Cross-Sectional Data)

When the need for supportive care for the different aspects of QOL is presented by year as shown in Figure 2, some general trends can be observed. First, the proportions with need for supportive care remain stable over time as can be seen for ‘global health status/QOL’, ‘social functioning’, ‘fatigue’, and ‘pain’. Second, an increase in the proportions with need for supportive care can be seen in the later years and especially during the pandemic era. This was true for ‘physical’ and ‘role functioning’, where the proportions diminished over time until the start of the pandemic, and increased subsequently (albeit slightly). It was also true for ‘emotional functioning’ and ‘insomnia’, where the proportion with need followed a U-shaped curve. Third, a decrease in the proportion with need for supportive care over time can be observed, which was especially true for ‘nausea and vomiting’, where the proportion with need showed a remarkable reduction during the pandemic era. However, the confidence intervals around the point estimators indicate a lack of statistical significance for the changes over time.

The COVID-19 pandemic which started in early 2020 can be regarded as a world health crisis, which has not been seen for over 100 years. Today, there is a vast majority of reports about the early effects of the pandemic on the societal and the individual level [23,24,25,26]. A decline in health-related QOL has been reported for the general population [45,46] as well as for vulnerable subgroups such as cancer patients [31,47,48,49]. In line with these reports, we observed that ‘emotional functioning’ is the QOL aspect with the highest proportion of need for supportive care during the pandemic era. In our cohort, ‘insomnia’ is the QOL domain with the second biggest proportion of need for supportive care, followed by ‘fatigue’, ‘social functioning’, ‘pain’, and ‘physical functioning’, with >70% in need for each. Additionally, but on a slightly lower level, the proportion with need for supportive care is highest during the pandemic era on the ‘global health status/QOL’ scale.

The lower proportion of women with need for supportive need with regard to ‘nausea and vomiting’ fits with the external evidence that cancer treatment and care in Germany altered with the start of the COVID-19 pandemic [28,29]. Over the entire pandemic period, provision of care was especially reduced in the areas of follow-up care (by 21% compared to pre-pandemic) and psycho-oncologic care (by 12%), with even higher reductions at the beginning of the pandemic in 03-04/2020 [28]. Anecdotal evidence from physicians in oncological clinics and in our rehab clinic indicates that in particular, different SACT regimes were given, while lymph drainage and physiotherapy was less often applied (unfortunately not seen in our data). However, comparisons of clinical characteristics and treatments of our study population between subgroups defined by time periods did not show substantial differences (Table 2 and Table 3), which could have otherwise explained changes in supportive care needs.

Given the limitation that data over time, as shown in Figure 2, are derived from different cross-sectional surveys with one measurement per woman rather than from a cohort with multiple ratings per woman (longitudinal data), it is conceivable that the general situation during the pandemic with lockdowns in Germany from March until May 2020 (closing down of nearly all stores, restaurants, interruption of sports and tourism, as well as social distancing), during November 2020 (“lockdown light”: social distancing), and from December 2020 until March 2021 (predominantly social distancing), which resulted in working from home where possible and the closing down of kindergartens and schools [50] as well as a general feeling of insecurity, including financial and economic losses, and an increase in worries (also COVID-19 pandemic-related fears) and mental burden [27,45,49,51], has contributed to the higher proportion with need for supportive care as observed for ‘emotional functioning’, ‘insomnia’, ‘fatigue’, ‘pain’, and ‘role functioning’. With the exception of the latter, where an increase of around 15% for the proportion with need was observed, changes were not regarded as clinically relevant.

The authors expected to find even more severe QOL restrictions with the start of the COVID-19 pandemic, as we have described the impact of the pandemic on mental health, and this seemed to apply especially to the younger age group of 18 to 35 years [51]. Since our BC patients with minor children showed very high proportions with need for supportive care independently from the COVID-19 pandemic, their level might not have risen as much as one would have expected. Two things could be the cause of this: (1) a “ceiling effect”—the values were already so high/low before the pandemic that a further increase in needs can no longer be mapped well via the questionnaire and/or the thresholds used, and (2) in our population of young BC patients with minor children participating in a 3-week inpatient rehab program, the effects of the pandemic on QOL were significantly less than the effects of facing the diagnosis and treatment of early-onset breast cancer.

### 4.3. Proportion with Need for Supportive Care at Baseline and Follow-Up (Longitudinal Data)

For a subgroup of 176 women, longitudinal data are available, as they participated in our survey twice. The baseline assessment was conducted approximately 12 months and the follow-up assessment approximately 32 months after diagnosis. Describing time trends with longitudinal data has some advantages (e.g., observing individual patterns of change), but longitudinal data may also be prone to selection bias. In our cohort, the women without follow-up information were more likely to have had locally advanced tumours (N0: 59% vs. 68% in those with follow-up), negative BC family anamnesis (54% vs. 47%), and triple-negative tumours (30% vs. 24%) at baseline compared to women who took part in our follow-up survey. Furthermore, women with follow-up information were more likely to have reported being single (19% vs. 8%), having low/intermediate education (63% vs. 54%), not disclosing working status (8% vs. 2%) or working as “workers” (11% vs. 6%), not disclosing income (8% vs. 2%), and having an income of EUR < 1500 (17% vs. 9%). Regarding treatment, women without follow-up information were more likely to have received radiotherapy (81% vs. 74%) and less likely to have received hormone therapy (57% vs. 69%). This selection of BC patients could have led to an overly positive evaluation of QOL several years after BC diagnosis.

The proportions with need for supportive care had decreased at follow-up for all QOL aspects. The largest differences were seen with regard to ‘role functioning’, ‘physical functioning’, and ‘social functioning’ (all with medium effect sizes). For the symptom scales/items, the differences were smaller and effect sizes were rather small. The proportions with need for supportive care due to ‘pain’, ‘fatigue’, and ‘sleep disturbance’ remained at a high level at follow-up. This is in line with Peters et al., where a large difference on the scale ‘role functioning’ was found when ratings before and after rehab were compared. This scale relates to tasks in everyday life (work, hobbies, etc.) and it is conceivable that, besides a general improvement in health status, patients found different ways to adapt over time. Raised levels of fatigue among BC patients and even (very) long-term survivors are a known problem [52,53], and it is conceivable that they are also associated with sleep disturbances.

Again, the proportions with need for supportive care at follow-up are much higher than those found by Lidington et al. [22], while the mean years from diagnosis are aligned between the Lidington et al. and our follow-up cohort. The authors propose that their patients—compared to patients shortly after diagnosis and primary treatment—had higher expectations concerning their QOL and functioning, now that their diagnosis was on average 2.8 years ago [22]. This implies that support needs in survivor populations are found at lower scores and less severe QOL scores than in populations currently undergoing treatment or shortly after treatment termination, which, in turn, could explain why our proportions were much higher in the baseline survey. However, if this had been the major factor, the proportions should have aligned in our follow-up cohort, which we did not observe.

### 4.4. Practical Implications for Provision of Supportive Care

While there is a specific guideline for supportive therapy (which targets side effects of oncological treatment) [54], the recommendations for supportive care remain quite vague in the evidence-based practice guidelines for breast cancer. Supportive care not only includes (1) educating the patient about social, financial, and psycho-oncological aspects of the disease, but also (2) providing physical, psychological, social, and spiritual support. Although the aforementioned guideline emphasizes the positive effects of counselling for patients and survivors, it neither states who is supposed to provide information nor when. However, the guideline recommends using validated questionnaires/measures to assess patients’ needs. In case of necessity, professional arrangements should be initiated. Cancer self-help groups are described to be helpful throughout the course of the disease (i.e., at diagnosis, during therapy and follow-up care [55]. In Germany, cancer counselling centres (German: “Krebsberatungsstellen”) in particular represent another important source for supportive care.

Although diverse counselling and supportive care possibilities exist, it must be assumed that many BC patients are not aware of them—maybe because they did not receive the respective information at all, they did not understand them as a consequence of lacking health literacy, or they did receive it, but at a time point (e.g., during their hospital stay for surgery) when it lacked relevance for them [56,57]. In particular, long-term effects or detriments persisting into long-term survivorship such as fatigue possibly need to be addressed at a later stage of the course of the disease in order to avoid an information overload of the patients during the acute phase of diagnosis and treatment initiation. An earlier survey among women in this rehabilitation program emphatically showed this lack of information: more than 4 out of 10 women had no knowledge about existing psychosocial support offers that include their family, albeit they were supposed to have been informed about it (according to the guideline) [10].

A lack of connection between different existing support offers in the German health system has been acknowledged, and model projects are planned to establish orientation guides and survivorship programs for long-term survivors. These are supposed to systematically bundle existing structures and initiate their use with the consideration of individual needs [57].

In summary, supportive care needs are common, but at the same time highly individual. Clinical guidelines should specify exactly when and who should (1) assess supportive care needs and (2) educate the patients regarding supportive care. Securing an information provision when patients transition from acute care to follow-up care and into long-term survivorship is essential, and the planned survivorship programmes are promising. Finally, the provision of supportive care needs to be tailored to the individual patient and should come from an interdisciplinary team including physicians, psychologists, nurses, and pain and sleep therapists.

### 4.5. Strengths and Limitations

A major strength of our study is the large sample size, with more than 800 women from all over Germany. Over a time period of 7 years, the same recruitment method was used to generate a sample, which was surveyed by a self-administered questionnaire. In principle, every BC patient with a child up to 12 years old could participate, but the rehab program is restricted to non-metastatic BC. Hence, the generalizability of our results is limited to these mothers with minor children and non-metastatic BC, as well as due to the aforementioned possible confounding by indication. Furthermore, the women in our study primarily belonged to the highest of the three income groups that we asked about in our questionnaire, which also limits the generalizability. However, when we started our study, the monthly mean net income in Germany was about EUR 3000. Since then, a rise has been observed and the mean net income equalled about EUR 3300 in 2016 and EUR 3800 in 2021 [58]. Thus, the category ’highest income’ of the questionnaire today refers to the mean income of the German population rather than that of well-off Germans.

The transfer of Lidington et al.’s thresholds also has some flaws, as our patients were surveyed shortly after their diagnosis and had received more treatment. To overcome this problem, we also analysed follow-up data. Furthermore, the application of thresholds derived from a non-German speaking sample could be questioned, but we believe that is reasonable to use them. Nolte et al. [59] demonstrated that mean values of the EORTC QLQ-C30 scales were quite comparable between the British and German general population, and furthermore, thresholds for clinical importance assessed in a large European study were quite robust and did not differ between the various regions within Europe [18]. Due to the data extraction from patient files, pandemic-related changes in treatment are overlooked; there is no information on lymph drainage nor whether treatment plans have been modified due to the pandemic. Additionally, a comparison of SACT over time is hampered by different data extraction practices.

## 5. Conclusions

Our findings point out that even several years after diagnoses, more than every second young BC patient with minor children has a need for supportive care. Contrary to our expectations, no remarkable increase in proportions with need was observed during COVID-19 pandemic. At the same time, it remains questionable whether an increase can be seen when the proportions are already this high in the beginning (“ceiling effect”). Our results are limited to a specific population and, therefore, further studies among young adult cancer patients (with and without minor children) using the thresholds by Lidington et al. [22] are needed to help with the comparison and interpretation of our results in a broader context. Preferably, these studies will provide age- and gender-specific results, as QOL is known to be dependent on those characteristics. Nevertheless, our results highlight the importance, first, of an early QOL screening among young adult BC patients with minor children and, second, of offering interventions for patients with need for supportive care not only during rehabilitation but also in the longer outpatient follow-up care. Existing interventions in this setting should be critically reviewed to determine whether they reach the patients in need.

## Figures and Tables

**Table 1:** Socio-demographic characteristics of young adult non-metastatic breast cancer patients with minor children participating in a 3-week inpatient mother–child rehab program according to time of rehab stay.

		2015–2017 (*n* = 388)	2018–March 2020 (*n* = 323)	July 2020–2021 (*n* = 142)
Age at diagnosis	(Mean (SD))	35.1 (3.1)	35.1 (3.1)	35.8 (2.6)
Months between diagnosis and rehab	(Mean (SD))	11.3 (3.6)	12.7 (3.6)	14.7 (4.2)
Patient has permanent partner	No	42 (10.8)	39 (12.1)	18 (12.7)
	Yes	328 (84.5)	274 (84.8)	118 (83.1)
	Unknown	18 (4.6)	10 (3.1)	6 (4.2)
Education ^1^	Low	18 (4.6)	19 (5.9)	7 (4.9)
	Intermediate	208 (53.6)	168 (52.0)	66 (46.5)
	High	157 (40.5)	131 (40.6)	69 (48.6)
	Unknown	5 (1.3)	5 (1.5)	0 (0.0)
Work situation	Worker	31 (8.0)	27 (8.4)	8 (5.6)
	Employee/ Civil servant	331 (85.3)	272 (84.2)	128 (90.1)
	Self-employed/ Freelancer	14 (3.6)	15 (4.6)	3 (2.1)
	Unknown	12 (3.1)	9 (2.8)	3 (2.1)
Income ^2^	EUR < 1500	53 (13.7)	48 (14.9)	10 (7.0)
	EUR 1500–3000	159 (41.0)	120 (37.2)	46 (32.4)
	EUR > 3000	162 (41.8)	145 (44.9)	85 (59.9)
	Unknown	14 (3.6)	10 (3.1)	1 (0.7)

Data are displayed as absolute and relative frequencies if not otherwise stated. ^1^ ‘low’—until grade 9 or 10 (compulsory); ‘intermediate’—until grade 10; ‘high’—until grade 12 or 13. ^2^ Income refers to the monthly net income of all household members after taxes and social contributions.

**Table 2:** Clinical characteristics of young adult non-metastatic breast cancer patients with minor children participating in a 3-week inpatient mother–child rehab program according to time of rehab stay.

		2015–2017 (*n* = 388)	2018–March 2020 (*n* = 323)	July 2020–2021 (*n* = 142)
Tumour size (TNM-T)	T in situ	15 (3.9)	23 (7.1)	4 (2.8)
	T0	90 (23.2)	104 (32.2)	60 (42.3)
	T1	158 (40.7)	129 (39.9)	51 (35.9)
	T2	107 (27.6)	56 (17.3)	22 (15.5)
	T3	15 (3.9)	7 (2.2)	4 (2.8)
	T4	1 (0.3)	2 (0.6)	1 (0.7)
	TX/unknown	2 (0.5)	2 (0.6)	0 (0.0)
Nodal status (TNM-N)	N0	257 (66.2)	232 (71.8)	103 (72.5)
	N1	94 (24.2)	67 (20.7)	26 (18.3)
	N2	25 (6.4)	13 (4.0)	9 (6.3)
	N3	8 (2.1)	2 (0.6)	4 (2.8)
	NX/unknown	4 (1.0)	9 (2.8)	0 (0.0)
Grading	G1	24 (6.2)	10 (3.1)	5 (3.5)
	G2	140 (36.1)	119 (36.8)	48 (33.8)
	G3	197 (50.8)	173 (53.6)	81 (57.0)
	GX/unknown	27 (7.0)	21 (6.5)	8 (5.6)
Tumour biology ^1^	Luminal A	50 (12.9)	30 (9.3)	17 (12.0)
	Luminal B/B1	96 (24.7)	99 (30.7)	43 (30.3)
	Luminal Her2+/B2	90 (23.2)	69 (21.4)	27 (19.0)
	Her2-like/non-luminal Her2+	30 (7.7)	28 (8.7)	6 (4.2)
	triple-negative/basal-like	99 (25.5)	84 (26.0)	41 (28.9)
	Unknown	23 (5.9)	13 (4.0)	8 (5.6)
Positive family anamnesis for BC	No	201 (51.8)	146 (45.2)	54 (38.0)
	Yes	152 (39.2)	109 (33.7)	54 (38.0)
	Unknown	35 (9.0)	68 (21.1)	34 (23.9)

Data are displayed as absolute and relative frequencies. ^1^ According to the St Gallen consensus conference definition [37].

**Table 3:** Treatment of young adult non-metastatic breast cancer patients with minor children participating in a 3-week inpatient mother–child rehab program according to time of rehab stay.

		2015–2017 (*n* = 388)	2018–March 2020 (*n* = 323)	July 2020–2021 (*n* = 142)
Mode of surgery	Breast-conserving treatment	214 (55.2)	174 (53.9)	74 (52.1)
	Mastectomy without reconstruction	45 (11.6)	38 (11.8)	17 (12.0)
	Mastectomy with reconstruction	126 (32.5)	105 (32.5)	45 (31.7)
	Other	1 (0.3)	4 (1.2)	2 (1.4)
	Had surgery, but no further information	2 (0.5)	2 (0.6)	4 (2.8)
Radiotherapy (RT)	No RT	85 (21.9)	87 (26.9)	30 (21.1)
	RT	299 (77.1)	233 (72.1)	107 (75.4)
	Unknown	4 (1.0)	3 (0.9)	5 (3.5)
Chemotherapy (CT)	No CT	36 (9.3)	32 (9.9)	12 (8.5)
	CT with HER-2 targeted therapy	94 (24.2)	88 (27.2)	32 (22.5)
	CT without HER-2 targeted therapy	246 (63.4)	200 (61.9)	96 (67.6)
	CT, medication acc. to study protocol/unknown substances	12 (3.1)	2 (0.6)	1 (0.7)
	Unknown	0 (0.0)	1 (0.3)	1 (0.7)
Hormone therapy (HT)	No HT	137 (35.3)	126 (39.0)	50 (35.2)
	HT with SERM ^1^	234 (60.3)	176 (54.5)	71 (50.0)
	HT with AI ^2^	11 (2.8)	14 (4.3)	13 (9.2)
	HT with GnRH ^3^	3 (0.8)	7 (2.2)	5 (3.5)
	HT with unknown substances	2 (0.5)	0 (0.0)	3 (2.1)
	Unknown	1 (0.3)	0 (0.0)	0 (0.0)

Data are displayed as absolute and relative frequencies. ^1^ SERM: selective oestrogen-receptor modulator, ^2^ AI: aromatase inhibitor, ^3^ GnRH: gonadotropin-releasing hormone.

**Table 4:** Absolute and relative frequencies of 176 young adult non-metastatic breast cancer patients with minor children participating in a 3-week inpatient mother–child rehab program with EORTC QLQ-C30 scores indicating a need for supportive care at baseline and follow-up according to thresholds proposed by Lidington et al. [22].

EORTC QLQ-C30 Scale	Values Indicating the Need for Supportive Care ^1^	Baseline (*n* = 176)	Follow-Up (*n* = 176)	Statistical Significance ^2^	Effect Size ^3^
Global Health Status/QOL	values < 71	105 (59.7)	95 (54)	Chi^2^(*df* 1) = 1.45; *p* = 0.229	−0.08 (−0.2; 0.04)
Physical Functioning	values < 97	135 (76.7)	115 (65.3)	Chi^2^(*df* 1) = 7.2; *p* = 0.007	−0.21 (−0.34; −0.08)
Role Functioning	values < 92	130 (73.9)	100 (56.8)	Chi^2^(*df* 1) = 11.05; *p* = 0.001	−0.21 (−0.31; −0.11)
Emotional Functioning	values < 71	151 (85.8)	139 (79)	Chi^2^(*df* 1) = 3.69; *p* = 0.055	−0.17 (−0.32; −0.01)
Social Functioning	values < 92	149 (84.7)	131 (74.4)	Chi^2^(*df* 1) = 6.02; *p* = 0.014	−0.19 (−0.32; −0.05)
Fatigue	values > 28	133 (75.6)	119 (67.6)	Chi^2^(*df* 1) = 3.36; *p* = 0.067	−0.17 (−0.32; −0.02)
Nausea and Vomiting	values > 8	37 (21)	30 (17)	Chi^2^(*df* 1) = 0.66; *p* = 0.417	−0.08 (−0.24; 0.09)
Pain	values > 8	128 (72.7)	122 (69.3)	Chi^2^(*df* 1) = 0.43; *p* = 0.511	−0.05 (−0.19; 0.08)
Insomnia	values > 17	134 (76.1)	121 (68.8)	Chi^2^(*df* 1) = 3.02; *p* = 0.082	−0.15 (−0.3; −0.01)

Displayed are absolute and relative frequencies. Only women who answered both the baseline and the follow-up questionnaire were included in the analysis. ^1^ According to Lidington et al. [22]. ^2^ McNemar test. ^3^ Cohen’s g (95% CI).

## Data Availability

The dataset of this study is not publicly available because informed consent from study participants did not cover the public deposition of data. The dataset is available from the corresponding author on reasonable request.

## Supplementary Materials

The following supporting information can be downloaded at: https://www.mdpi.com/article/10.3390/cancers15061770/s1, Table S1: Mean EORTC QLQ-C30 scores of young adult non-metastatic breast cancer patients participating in a 3-week inpatient mother–child rehab program according to the time of rehab stay. Table S2: Measures of internal consistency, number of items contributing to the EORTC QLQ-C30 scales, and information on whether threshold for need of supportive has been published for young cancer patients. Table S3: Absolute numbers and relative proportions of women with need for supportive care at follow-up for young adult non-metastatic breast cancer patients participating in a 3-week inpatient mother–child rehab program (cohort 4) according to time difference between diagnosis and follow-up survey in the year 2018.
