# Supplementary material for: Young Adult German Breast Cancer Patients Participating in a Three-Week Inpatient Mother–Child Rehab Program Have High Needs for Supportive Care
Source: Cancers (Basel). 2023 Mar 15;15(6):1770. doi: 10.3390/cancers15061770 (PMC10046589; doi:10.3390/cancers15061770)
Supplement: Supplementary file 1 [file cancers-15-01770-s001.zip › Table S1 Mean EORTC QLQ-C30 scores with p-values and effect sizes.pdf]

Supplementary Table S1: Mean EORTC QLQ-C30 scores of young adult non-metastatic breast cancer patients with minor children participating in a 3-week inpatient mother-child rehab program according to the time of rehab stay (Mean (SD)).

**EORTC QLQ-C30 scale**	**2015**	**2016**	**2017**	**2018**	**2019**	**Jan. -March 2020**	**July-Dec. 2020**	**2021**	**Statistical significance** F-Test from ANOVA	**Effect size** Omega squared (95-% CI)
Global Health Status/QOL	65.4 (16.5)	65.9 (16)	67.6 (16)	66 (17.2)	64.9 (18.7)	65.6 (18.3)	65.3 (14.4)	64.6 (17.4)	F(*df* 1, 842) = 0.79, p = 0.347	0 (0; 1)
Physical Functioning	78.6 (16.2)	82.3 (16.5)	82.6 (15.7)	82.7 (16.5)	82.2 (16.9)	85.4 (17.5)	82.9 (17.4)	85.6 (15)	F(*df* 1, 844) = 5.21, p = 0.023	0 (0; 0.02)
Role Functioning	63.5 (28)	66.1 (29.5)	68.9 (28.1)	67.4 (30.1)	66.4 (30.7)	75 (29.7)	72.4 (29.7)	70.3 (28.1)	F(*df* 1, 842) = 3.0, p = 0.084	0 (0; 0.01)
Emotional Functioning	43.8 (24.1)	47 (22.8)	50.5 (26.5)	48.6 (27.7)	47.6 (27.9)	44.8 (29.9)	50.2 (22.7)	47.4 (22.1)	F(*df* 1, 846) = 0.08; p = 0.775	0 (0; 1)
Cognitive Functioning	48.7 (31.4)	59.6 (28.8)	58.9 (30.9)	57.4 (31.7)	63.1 (27.5)	57.9 (30.4)	57.7 (25.6)	61 (25.3)	F(*df* 1, 848) = 3.14; p = 0.077	0 (0; 0.01)
Social Functioning	56.9 (29.2)	57.4 (27.6)	59.8 (30.7)	57.9 (32.1)	54.5 (31.6)	60.1 (31.8)	60.4 (28.2)	66.3 (27.9)	F(*df* 1, 844) = 2.44; p = 0.118	0 (0; 0.01)
Fatigue	55.2 (29.9)	49.6 (29.1)	49.2 (26.5)	51.6 (27.7)	48.7 (26.8)	49.1 (28.3)	47.8 (23.4)	46.4 (25.2)	F(*df* 1, 844) = 2.49; p = 0.115	0 (0; 0.01)
Nausea and Vomiting	10.9 (20.8)	6.4 (14.5)	8.3 (17.5)	8.5 (17.4)	6.9 (15.4)	7.9 (19.3)	6.7 (13.5)	4.7 (14.8)	F(*df* 1, 844) = 2.81; p = 0.094	0 (0; 0.01)
Pain	37 (29.9)	31.5 (27.8)	35.3 (28.5)	36.7 (30.4)	36 (28.9)	25.4 (27.3)	38.6 (28.8)	30.2 (26.4)	F(*df* 1, 844) = 0.65; p = 0.419	0 (0; 1)
Dyspnoea	36.5 (36)	29 (32.2)	31.3 (30.2)	29.9 (32.5)	29.3 (33.2)	20.2 (23.9)	26.5 (28.2)	30.6 (30.5)	F(*df* 1, 841) = 1.79; p = 0.181	0 (0; 0.01)
Insomnia	53.1 (35)	45.5 (37.3)	47.7 (36.4)	50 (35.7)	47.1 (36.6)	54.1 (34.6)	59 (35.3)	53.1 (33)	F(*df* 1, 839) = 2.08; p = 0.150	0 (0; 0.01)
Appetite loss	16.1 (30.9)	9 (21.1)	11.4 (21.7)	17.9 (28.9)	9 (17.1)	7 (15.8)	8.6 (18.7)	9.7 (20.5)	F(*df* 1, 843) = 1.75; p = 0.186	0 (0; 0.01)
Constipation	21.4 (34.3)	14.5 (26.9)	12.6 (25.4)	16.2 (28.2)	10.8 (25.2)	14.9 (30.7)	19 (34.6)	13.7 (25.5)	F(*df* 1, 843) = 0.76; p = 0.382	0 (0; 1)

Diarrhoea	11.1 (23.2)	6.7 (16.1)	9.9 (21.6)	11.2 (25.4)	13.5 (26.7)	9.6 (25.6)	9.5 (20.7)	10.2 (23.4)	F(*df* 1, 839) = 0.96; p = 0.328	0 (0; 1)
Financial Difficulties	42.1 (40.9)	37.4 (35.8)	33.3 (34.8)	37.3 (35.4)	41.1 (34.8)	39.5 (36.2)	27.9 (35.6)	23.7 (29.2)	F(*df* 1, 838) = 6.18; p = 0.013	0 (0; 0.02)
