# Supplementary material for: Young Adult German Breast Cancer Patients Participating in a Three-Week Inpatient Mother–Child Rehab Program Have High Needs for Supportive Care
Source: Cancers (Basel). 2023 Mar 15;15(6):1770. doi: 10.3390/cancers15061770 (PMC10046589; doi:10.3390/cancers15061770)
Supplement: Supplementary file 1 [file cancers-15-01770-s001.zip › Table S2 measures of internal consistency_EORTC.pdf]

Supplementary Table S2. Measures of internal consistency, number of items contributing to the EORTC QLQ-C30, and information whether threshold for need of supportive has been published for young cancer patients

EORTC QLQ-C30 scale	Number of items in scale	Measure of internal consistency[2]	Thresholds for need of supportive care available[1]
Global Health Status/QOL	2	0.83	yes
Physical Functioning	5	0.71	yes
Role Functioning	2	0.85	yes
Emotional Functioning	4	0.87	yes
Cognitive Functioning	2	0.72	no
Social Functioning	2	0.85	yes
Fatigue	3	0.88	yes
Nausea and Vomiting	2	0.67	yes
Pain	2	0.86	yes
Dyspnoea	1	n.a.	no
Insomnia	1	n.a.	yes
Appetite loss	1	n.a.	no
Constipation	1	n.a.	no
Diarrhoea	1	n.a.	no
Financial difficulties	1	n.a.	no

[1] According to Lidington et al 2022.
[2] In case of two items Spearman-Brown-coefficient, in case of three or more items Cronbach's alpha
n.a. not applicable.
