# Supplementary material for: Young Adult German Breast Cancer Patients Participating in a Three-Week Inpatient Mother–Child Rehab Program Have High Needs for Supportive Care
Source: Cancers (Basel). 2023 Mar 15;15(6):1770. doi: 10.3390/cancers15061770 (PMC10046589; doi:10.3390/cancers15061770)
Supplement: Supplementary file 1 [file cancers-15-01770-s001.zip › Table S3 need for supp.care at follow up with p values effect size.pdf]

Supplementary Table S3. Absolute numbers and relative proportions of women with need for supportive care at follow up of young adult non-metastatic breast cancer patients participating in a 3-week inpatient mother-child rehab program (cohort 4) according to time difference between diagnosis and follow up survey in the year 2018.

EORTC QLQ-C30 scale	Values indicating the need for supportive care[1]	<= 24 months (n=28)	25-36 months (n=91)	>36 months (n=57)	Statistical significance[2]	Effect Size[3]
Global Health Status/QOL	value <71	15 (53.6)	52 (57.1)	28 (49.1)	$Chi^2(df\ 2) = 0.91, p = 0.635$	0.07
Physical Functioning	value <97	19 (67.9)	60 (65.9)	36 (63.2)	$Chi^2(df\ 2) = 0.33, p = 0.849$	0.04
Role Functioning	value <92	20 (71.4)	50 (54.9)	30 (52.6)	$Chi^2(df\ 2) = 2.84, p = 0.242$	0.13
Emotional Functioning	value <71	26 (92.9)	69 (75.8)	44 (77.2)	$Chi^2(df\ 2) = 3.90, p = 0.142$	0.15
Social Functioning	value <92	24 (85.7)	65 (71.4)	42 (73.7)	$Chi^2(df\ 2) = 2.13, p = 0.345$	0.11
Fatigue	value >28	21 (75.0)	59 (64.8)	39 (68.4)	$Chi^2(df\ 2) = 0.75, p = 0.688$	0.07
Nausea and Vomiting	value >8	5 (17.9)	15 (16.5)	10 (17.5)	$Chi^2(df\ 2) = 0.02, p = 0.990$[4]	0.01
Pain	value >8	21 (75.0)	64 (70.3)	37 (64.9)	$Chi^2(df\ 2) = 0.99, p = 0.610$	0.07
Insomnia	value >17	20 (71.4)	57 (62.6)	44 (77.2)	$Chi^2(df\ 2) = 2.89, p = 0.24$	0.13

[1] According to thresholds proposed by Lidington et al 2022; [2] Chi-squared test ; [3] Cramer's V; [4] Due to low number of expected cases, results from Chi-squared test may be flawed
